# Material property changes during electrohydrodynamic (EHD) drying a closer look into the falling rate period

**DOI:** 10.1016/j.crfs.2025.101181

**Published:** 2025-08-20

**Authors:** Zulhaj Rizki, Judith C.A. Ham, Remko M. Boom, Maarten A.I. Schutyser

**Affiliations:** Laboratory of Food Process Engineering, Wageningen University & Research, Bornse Weilanden 9, 6408 WG, Wageningen, the Netherlands

**Keywords:** Electrohydrodynamic, Drying, Modeling, Multiphysics model, Corona wind

## Abstract

Electrohydrodynamic (EHD) drying offers an alternative to hot air drying thanks to its lower energy use and lower operating temperatures. EHD drying benefits from enhanced convection induced by the corona wind, but during the falling-rate period is constrained by internal moisture transport. It has recently been hypothesized that the internal transport can be enhanced since the product is subjected to an electric field.

This paper combines a theoretical analysis and experimental characterization to quantify the evolution of the electric potential across a thin layer during EHD drying of porous materials. Results reveal that during the constant-rate drying period, the electric potential remains low, confirming that drying is predominantly controlled by corona wind-driven convection. As drying progresses to the falling-rate period, the electric potential over the layer increases, which may facilitate electromigration. However, the material's electric resistance simultaneously increases due to reduced moisture content, which may counteract this.

The quantification of the electric potential and material permittivity during EHD drying, advances our understanding of electrically driven mass transport, and will allow us to better model electrically-driven processes and drying, supporting the development of optimized EHD drying technologies.

## Highlights

• Residual potential in EHD drying is quantified using multiple methods.
• Low electric potential during constant drying shows limitation by corona wind.
• Electric potential increase later in drying may induce electrically driven effects.
• Increased resistance following the rise of electric potential counteracts the effect.

## Graphical abstract

**Figure:**
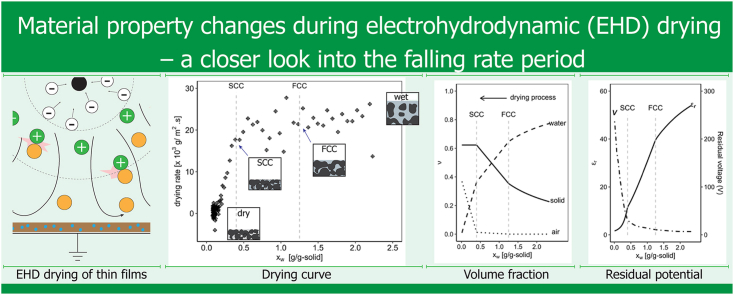

## 1 Introduction

Electrohydrodynamic (EHD) drying is emerging as a milder alternative to conventional air-drying processes. EHD-drying makes use of a high electric field to create a corona wind (Wang et al., 2020), which is then used for drying. It has been reported that EHD drying enhances the mass transfer thus allowing a faster drying kinetics (Defraeye and Martynenko, 2019; Defraeye and Martynenko, 2018a; Martynenko and Misra, 2021). This improves the efficiency of drying and can reduce the energy consumption (Rubinetti et al., 2024; Onwude et al., 2021; Martynenko et al., 2021; Raghavan et al., 2005). Faster drying allows the use of lower temperatures, making EHD drying suitable for mild drying of heat-sensitive materials such as food.

Although EHD has been investigated for quite some time, its application in drying processes emerged much later (Singh et al., 2012; Kulacki and Daumenmier, 1978). To date, no larger scale implementations have been reported, primarily due to an incomplete understanding of the process itself. Various studies successfully demonstrated this drying method for various materials, especially food—including fruit slices (Paul et al., 2022; Bardy et al., 2016), carrots (Ding et al., 2015; Wang and Ding, 2023; Alemrajabi et al., 2012), and shrimps (Bai and Sun, 2011). Studies have been carried out to improve the understanding of this process via both experimental and modeling approaches (Defraeye and Martynenko, 2019; Onwude et al., 2021; Wang and Ding, 2023; Taghian Dinani et al., 2014; Defraeye and Martynenko, 2018b; Iranshahi et al., 2022).

Based on our current understanding, EHD drying involves two sequential phenomena: the generation of corona wind and the subsequent drying process. EHD drying is fundamentally a convective air drying process where heat and mass transfer is enhanced by the impinging corona wind. However, during EHD the product is positioned between the emitter and collector electrodes, exposing it to the electric field. This exposure has led to the hypothesis that an electrically driven mechanism may also contribute to the drying process. A recent review by Ham et al. (2024) summarizes the electrically driven phenomena that may internally enhance the drying process.

To accurately describe electrically-driven phenomena during the drying process, it is important to understand the residual electric potential and current in the product as a function of the moisture content. This electric potential across the product layer is the main driving force for electrically driven transport. One may estimate the electric driving force by using the applied potential difference between the electrodes, but the potential diminishes rapidly with the distance from the emitter, and it is not clear what fraction of the electric potential acts over the product layer. Directly measuring electric potential on the products remains practically challenging.

Modeling can be a better alternative for estimating the residual electric potential across drying materials. A recent study demonstrated that the residual potential in thin layers during EHD is influenced by the material's permittivity (Robinson et al., 2002). In an electric field, materials with a higher permittivity generally induce stronger polarization of their molecular dipoles, thereby reducing the internal electric field. Consequently, materials with a higher permittivity tend to exhibit a lower residual potential. Permittivity data for various food materials at different moisture levels have been reported in multiple independent studies (Robinson et al., 2002; Tıraş et al., 2019; Icier and Baysal, 2004). However, a direct correlation between the two has not yet been established for these materials. In contrast, such a relationship has been the subject of several studies in soil science (Szypłowska et al., 2021; Tabbagh et al., 2000).

In this study, we integrate multiple theoretical approaches and experimental characterization to establish this critical relationship. By doing so, we aim to estimate the residual potential during drying, which can subsequently be linked to electrically driven phenomena, such as electroosmosis (Ham et al., 2024; O'Brien, 1986) and Ohmic heating (Golsefid et al., 2017; Astráin-Redín et al., 2024).

To integrate electrically driven phenomena into the EHD framework, it is crucial to describe the residual potential over the material layer, as this serves as the driving force for such processes. In a previous study (unpublished work) we established a relationship between the material's permittivity and the residual potential observed in thin layers within an EHD dryer. This residual potential is significantly lower than the applied potential but is considered the actual driving force behind electrically driven phenomena within the drying film in EHD drying. Despite its importance, this parameter has not been addressed in other studies. Building on these earlier findings, the present research aims to correlate moisture content with permittivity in order to simulate changes in residual potential across a thin layer during the EHD process. The analysis focuses on drying of a starch layer model system representing granular insoluble solids or porous media, which are commonly found in foods.

## 2 Material and methods

### 2.1 Materials

The model material used in this study is potato starch powder obtained from Sigma-Aldrich, Germany. The true density of starch powder was characterized using UltraPyc 1200e density meter (Anton Paar, Austria) with nitrogen as the inert gas. The particle size distribution of starch powder was characterized at 2 bar using Malvern Mastersizer 3000 (Malvern PANalytical, UK).

### 2.2 EHD drying experiments

Experiments were performed to characterize the starch powder as well as its drying behaviour within an EHD drying set up. MilliQ® water was added to the powder to obtain 70 %-wet basis water content (2.3 g-water/g-solid). This initial water content was applied to all drying experiments.

Electrohydrodynamic (EHD) drying experiments were conducted using a custom-built setup. The system employed a wire-to-plate electrode configuration, with a wire emitter of 100 μm diameter and a stainless-steel plate serving as both the collector electrode and the sample holder. A potential of 17 kV was applied to the emitter electrode, while the collector electrode was grounded. The selected potential difference was based on the stability observed during preliminary trials. The sample holder was connected to a balance, enabling real-time monitoring of the sample weight, which was recorded at 6-s intervals. Three EHD experiments were performed to analyse the drying curves using three different conditions: at 20 °C without electricity (reference with natural convection), at 20 °C with applied Voltage of 17 kV, and at 40 °C with applied Voltage of 17 kV. The experiments were conducted using 3 g of a starch suspension in a 10 × 12 cm holder.

## 3 Results and discussion

**Fig. 1:**
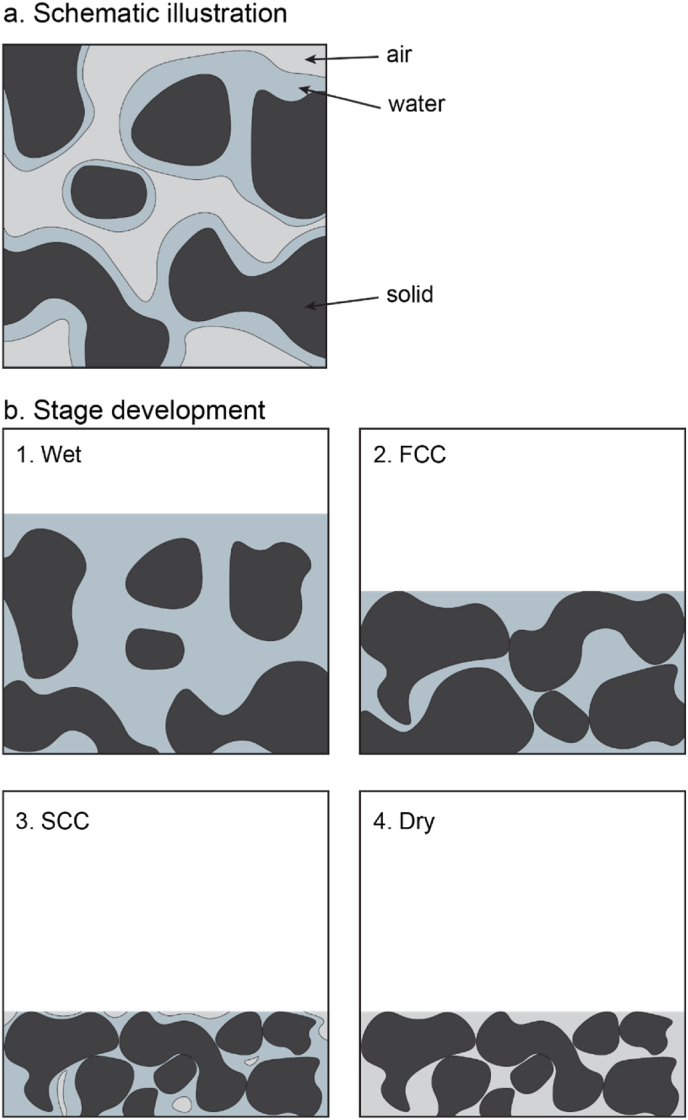
(a) Schematic illustration and (b) Stage development of 3-phase model system for moist layer during drying following 4 conditions: 1. Wet, 2. First Critical Condition (FCC), 3. Second Critical Condition (SCC) and 4. Dry.

A moist thin layer of a product like starch can be modelled as a three-phase system comprising solid material, water, and air (Fig. 1). The layer's permittivity can be estimated using a mixture theory, which uses the volume fraction of each component for finding the effective permittivity. Consequently, understanding how the volume fractions of these components evolve during the drying process is essential for accurately addressing this challenge.

### 3.1 The product layer as a 3-phase system

It is assumed that the internal pores within the solid particles are minimal or non-existent, and that their contribution to the drying dynamics is negligible. The solid particles are dispersed within either the water or the air phase, depending on the stage of the drying process. Initially, the layer behaves like a wet paste, with water serving as the continuous phase that suspends the solid particles. It is assumed that at the start of drying no air bubbles are present. During drying, air will gradually penetrate the cavities between the solid particles. The transitional behaviour between these stages is unclear.

Watanabe and Lepoutre (1982) identified three distinct stages of drying for thin layers, characterized by two critical conditions marking the transitions between stages. This 3-phases thin layer drying mechanism proposed by Watanabe and Lepoutre has been widely adopted in various studies (Bernada and Bruneau, 1997; Eckersley and Rudin, 1994; Younis et al., 2001; Laudone et al., 2006; Laudone et al., 2004). In the first stage, the layer behaves as a slurry (Fig. 1b.1), with water freely available throughout this phase. During this stage, water evaporates easily from the layer's surface without any significant resistance. The drying is initially governed primarily by external factors, namely the convective transport, rather than transfer limitations within the layer. This stage corresponds to the commonly recognized *constant drying rate period* in the general drying process.

During the constant drying period, water evaporates while the solid content remains unchanged, causing the layer to shrink as the water volume decreases. At this stage, the solid particles do not yet because the surrounding water is still abundant. However, as the water content diminishes, the particles rearrange into a more compact structure. Eventually, the surface particles lock into place, creating resistance to moisture transport. This corresponds to the locking point in many drying processes. A moisture gradient now starts to develop in the film that hinders diffusion of water to the surface and therefore the drying rate decreases. This transition into the falling rate period is marked by the first critical condition (FCC, Fig. 1b.2).

After the FCC, free water becomes scarce and the swollen particles begin to shrink, as they lose water. The inter-particle locking that occurs at the FCC is relatively mild, allowing the particles to continue restructuring in response to the shrinkage. As water evaporates, air starts intruding into the voids between particles, replacing the lost moisture and initiating the formation of pores. These processes occur simultaneously upon entering the falling rate period. Although the drying rate decreases, the reduction is not drastic, as the restructuring of the shrinking particles may occasionally free some of the water.

The processes of particle shrinking, restructuring and pore formation continue until the particles reach their final size, beyond which no further shrinkage is possible. At this stage, inter-particle locking becomes more pronounced, significantly increasing the resistance to moisture transport. This condition is identified as the second critical condition (SCC, Fig. 1b.3). Beyond the SCC, the layer structure becomes fully stabilized, while water evaporation persists. However, evaporation is now internally limited, as the free water on the layer surface is nearly depleted. Internal water transport, driven by diffusion and capillary action, is much slower than surface evaporation. Consequently, the drying rate drops drastically, marking the onset of the second falling rate period. Under ideal conditions, all water is eventually removed, albeit at a very slow rate, leaving the layer as a densely packed bed of solid particles (Fig. 1b.4).

One effective way to identify the critical points—FCC and SCC—is by analysing the drying curve. The drying curve illustrates the relationship between the drying rate and moisture content, $x_{w}$. over the process. If the drying process adheres to Watanabe and Lepoutre's proposed mechanism, the curve should exhibit three distinct trends corresponding to the three drying stages: the constant drying rate period, the first falling rate period, and the second falling rate period. The critical points, FCC and SCC, can be observed as transition points between these regimes. By carefully analysing these transitions in the drying curve, we can pinpoint the critical conditions that define the drying stages and gain insight into the drying behaviour of the material.

**Fig. 2:**
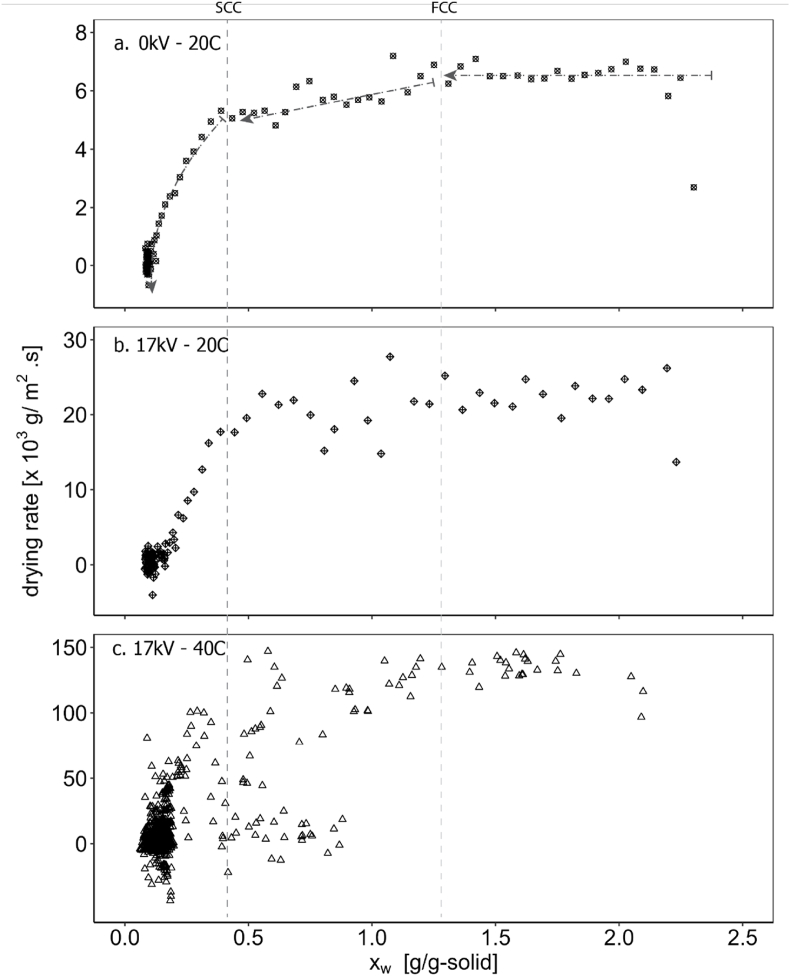
Drying curve of starch thin layer using the EHD drying set up under 3 different process conditions: (a) without electricity at 20 °C, (b) with 17 kV at 20 °C, and (c) with 17 kV at 40 °C. Dash-dot lines are drawn to guide the eyes.

We analysed the drying curve of a starch layer using an EHD drying setup and indeed observed three distinct regimes, confirming the three-stage drying mechanism proposed by Watanabe and Lepoutre. As shown in Fig. 2a, the initial phase exhibits constant drying where water evaporation is externally controlled. This stage continues until the FCC is reached. Following the FCC, the drying rate decreases linearly, marking the first falling rate period, which persists until the SCC. Beyond the SCC, the drying rate experiences a sharp and nonlinear decline. This drastic drop indicates that internal limitations such as diffusion and capillary transport, dominate the drying process during the final stage. These observations provide clear evidence of the three-stage mechanism and the critical transitions in the drying behaviour of starch layer.

Interestingly, we observed similar critical points (FCC and SCC) under three different drying conditions. As shown in Fig. 2a–c, both the FCC and SCC correspond to the same moisture content across all conditions, regardless of variations in the drying rate. This suggests that the critical conditions are intrinsic to the material and independent of the specific drying process parameters, within the given range of process parameters. The likely explanation is that these critical points depend on the material's inherent ability to rearrange and align its particles during the drying process. This is governed by the physical and chemical properties of the material itself. In contrast, the rate at which these transitions occur—reflected in the magnitude of the drying rate—is externally determined by the process conditions, such as temperature, humidity, or the drying setup. This highlights the material-specific nature of critical conditions while emphasizing the role of external factors in influencing the overall drying dynamics. It is important to note that this independence was observed under slow drying conditions and based on an average within a limited sample. At a more extreme drying rate, the critical points may change, as certain areas may dry faster than others.

Fig. 2 contributes to the limited data on EHD drying, enriching our knowledge on this technique. Fig. 2b indicates that EHD drying at 17 kV significantly has a significantly enhanced drying rate, achieving nearly three times the rate of the base scenario (Fig. 2a): the EHD constant drying rate was approximately 24 · 10^3^ *g/m*^*2*^ *s* compared to 6.5 · 10^3^ *g/m*^*2*^ *s* without electric field. When EHD was combined with a somewhat higher temperature of 40 °C, the drying rate improved further, reaching 140 mg/min. However, the experimental noise was considerably higher in the 40 °C drying experiments. This variability likely stemmed from the limitations in the experimental setup's ability to homogeneously heat the ambient air to 40 °C. Local temperature gradients may have formed, causing pressure differences that impacted the scale readings. Negative rate values observed in the drying curve are also believed to result from similar measurement noise. Such discrepancies were less pronounced in the experiments conducted at 20 °C. Despite these challenges, the results highlight the potential of EHD drying, particularly when combined with mild temperature elevation, to significantly accelerate drying rates.

In conclusion, we determined the critical moisture contents for the starch layer to be 1.25gwater/gsolid at the FCC and 0.4gwater/gsolid at the SCC.

### 3.2 Volume fraction formulation

As water evaporates during drying, pores develop, which leads to changes in the layer's composition. To estimate the properties of the layer as a mixture, the volume fraction of each component must be determined. The change in volume fraction during drying is governed by the drying mechanism. With the three-stage mechanism confirmed and the critical points identified, we can calculate the volume fractions of each component—solid, water, and air—at every drying stage. These calculations are based on definitions, including density $\left(\rho_{s,w,a}=\frac{m_{s,w,a}}{V_{s,w,a}}\right)$, moisture content $\left(x_{w}=\frac{m_{w}}{m_{s}}\right)$, and volume fraction $\left(\nu_{s,w,a}=\frac{V_{s,w,a}}{V_{s}+V_{w}+V_{a}}\right)$. A key consideration is that the total volume fraction must always sum to unity $\left(\nu_{s}+\nu_{w}+\nu_{a}=1\right)$. For simplicity, we assume ideal wet and dry conditions: no air is present in the wet slurry ($\nu_{a}^{wet}=0$), and no water remains in the dry layer ($\nu_{w}^{dry}=0$). Using these definitions and assumptions, we formulate the volume fractions for each drying stage. Table 1 provides an overview of the changes in the distribution of solid, water, and air throughout the drying process.

**Table 1:** Volume fraction formulation for each drying stage and critical condition.

	Wet	FCC	SCC	Dry
$\nu_{s}$	$\frac{1}{1+x_{w}\cdot \left(\frac{\rho_{s}}{\rho_{w}}\right)}$	$\frac{1}{1+x_{w}^{FCC}\cdot \left(\frac{\rho_{s}}{\rho_{w}}\right)}$	$\nu_{s}^{dry}$	$\nu_{s}^{dry}$
$\nu_{w}$	$1-\nu_{s}^{wet}$	$1-\nu_{s}^{FCC}$	$x_{w}^{SCC}\cdot v_{s}^{dry}\cdot \left(\frac{\rho_{s}}{\rho_{w}}\right)$	0
$\nu_{a}$	0	0	$1-\nu_{s}^{SCC}-\nu_{w}^{SCC}$	$1-\nu_{s}^{dry}$

In addition to the critical moisture contents determined in the previous section, solving the equations in Table 1 requires data on the density and the volume fraction in the dry state. The density of water is used as a standard reference. The density of the solid starch particles was measured in the laboratory, yielding a value of $1.4646\;\pm \;0.0014\;g/{cm}^{3}$. The volume fraction in the dry state can be estimated using the packing density theory for granular solids. Beyond the SCC, as water continues to evaporate, air progressively fills the void spaces between particles. It is reasonable to assume that, in the fully dry state, the particles achieve their maximum random packing density, representing the densest possible arrangement of the solid phase. This assumption provides a practical basis for calculating the volume fraction in the dry condition.

**Fig. 3:**
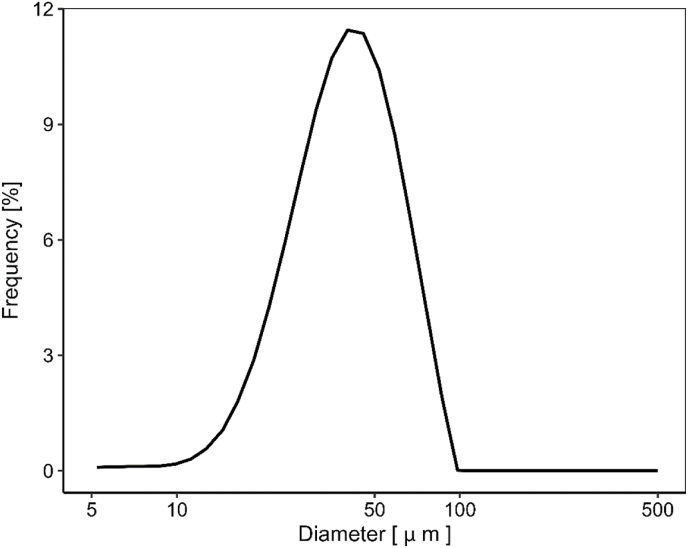
Particle size distribution of starch granules with volume-based frequency.

The packing density of monodispersed particles can be calculated geometrically based on their uniform size and arrangement. In contrast, polydisperse systems often exhibit higher packing densities due to the ability of smaller particles to fill the voids between larger ones. To estimate the packing density of polydisperse particles, mathematical models have been based on size distribution data, such as those proposed by Hao et al. (Hao and Riman, 2003) and Desmond et al. (Desmond and Weeks, 2014). The packing density estimates should align with the assumptions outlined within the equations in Table 1, particularly that the sum of all volume fractions must equal to unity. To support these calculations, we measured the size distribution of the starch particles, which is presented in Fig. 3.

**Fig. 4:**
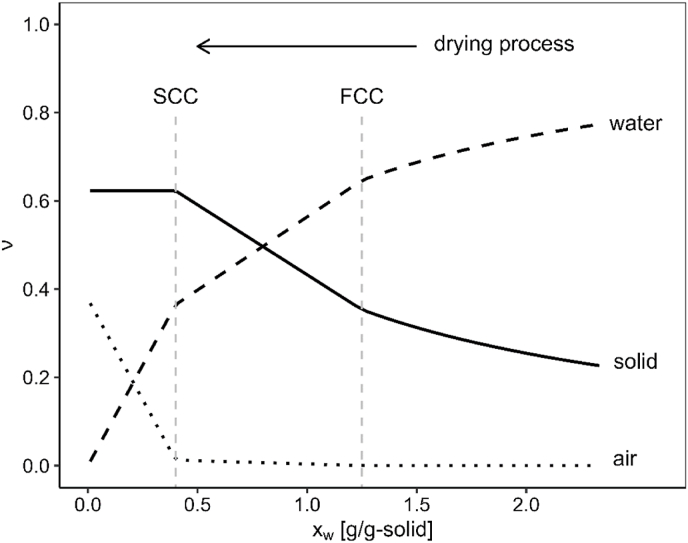
Evolution of the volume fractions of solid, water, and air throughout the three stages of the drying process.

Using the particle size distribution data and a mathematical description for the size distribution of polydisperse loose random packing developed by Desmond et al. (Desmond and Weeks, 2014), we were able to solve the system of equations summarized in Table 1. By applying an interpolation technique to estimate the volume fractions between the critical points, we calculated the complete evolution of volume fractions throughout the drying process. This is presented in Fig. 4.

During the first stage of drying, the constant drying rate period, the water fraction decreases steadily due to evaporation, while the solid fraction increases correspondingly. At this stage, there is no air present in the layer, as the system remains fully saturated with water. In the first falling rate period, the water fraction continues to decline, and the solid fraction keeps increasing, but at a slightly higher rate compared to the constant drying period. This is due to the intrusion of air, which begins to form small pores within the layer. At this stage, the amount of air remains relatively low, and the changes in volume fractions are moderate. In the final stage, the second falling rate period, the air fraction increases more rapidly as it replaces the remaining water. The water fraction eventually reaches zero, signifying the end of the drying process. During this stage, the solid fraction remains constant because the structure has stabilized at the SCC, and further rearrangement of particles is no longer possible.

### 3.3 Electric permittivity and residual potential estimation

With known volume fractions (Table 1), we can estimate the electric permittivity of a starch layer using theoretical mixture models. These models predict the effective permittivity of composite materials using the permittivity and volume fractions of the individual components. The permittivity values of air ($\varepsilon_{r,a}$ = 1) and water ($\varepsilon_{r,w}=80$) are well-documented in the literature and are used here as reference values. The permittivity of the dried solid phase, however, is not explicitly available in the literature. Nonetheless, studies on various dried food powders report permittivity values around 2 (Tıraş et al., 2019; Frabetti et al., 2023), leading us to approximate the solid permittivity as $\varepsilon_{r,s}\approx 2$ for this analysis. The evolution of the volume fraction of each component during drying has been determined and is presented in Fig. 4. By integrating these data with mixture theories, the progression of the layer's effective permittivity during the electrohydrodynamic (EHD) drying process can be estimated, offering insights into its changing electrical properties.

Theoretical models to describe the material properties of multiphase systems have been proposed in literature, including the Random Heterogeneous Media theory (Torquato, 1991), the Effective Medium Theory (EMT) (Nayak et al., 2023), the Percolation Theory (Leuenberger, 1999), the Maxwell-Garnett theory, and the Bruggeman theory (Markel, 2016). These models have been extensively validated and applied to a wide range of cases to estimate mixture properties. All these theories establish relationships between the volume fractions of each component in the mixture and their pure permittivity values. However, the specific relationship depends on the spatial distribution of each material within the mixture. For instance, Random Heterogeneous Media models incorporate detailed microstructural features using statistical correlation functions, making them suitable for complex systems. Simpler approaches, like EMT or the Maxwell-Garnett equation, are effective for dilute mixtures with weak interactions among components. Percolation Theory focuses on connectivity and critical thresholds, capturing nonlinear behaviour near phase transitions. Among these methods, the Bruggeman equation offers a versatile yet straightforward framework by symmetrically treating all phases, making it particularly well-suited for systems with multiple components. In our analysis, we utilized the Bruggeman equation (Eq. (1)) to estimate the effective permittivity of the thin layer, $\varepsilon_{f}$, leveraging its balance of simplicity and versatility.

(1) $$\nu_{s}\frac{\varepsilon_{s}-\varepsilon_{f}}{\varepsilon_{s}+2\varepsilon_{f}}+\nu_{w}\frac{\varepsilon_{w}-\varepsilon_{f}}{\varepsilon_{w}+2\varepsilon_{f}}+\nu_{a}\frac{\varepsilon_{a}-\varepsilon_{f}}{\varepsilon_{a}+2\varepsilon_{f}}=0$$

This equation expresses an implicit relationship of the film permittivity, $\varepsilon_{f}$, as a function of the permittivities of the individual phase: solid, $\varepsilon_{s}$, water, $\varepsilon_{w}$, and air, $\varepsilon_{a}$. The contribution of each component is proportional to its volume fraction, ν. By combining the prediction of the volume fractions (Fig. 4) with the permittivities of the individual phases as a function of time, the permittivity during drying can be calculated by solving Equation (1).

**Fig. 5:**
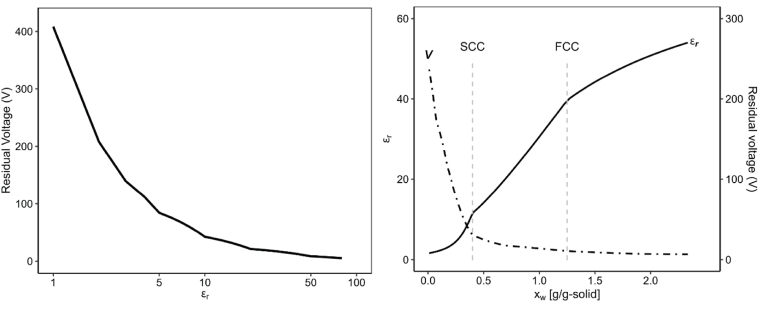
(a) Residual potential as a function of layer permittivity simulated by a Multiphysics model using layer thickness of 0.6 mm, and (b) permittivity and residual potential evolution within a thin layer during EHD drying.

The relationship between layer permittivity and the residual potential across the layer was demonstrated in a previous simulation study using a Multiphysics model (unpublished results), as summarized in Fig. 5 a. In this simulation, the lower part of the film is grounded, and the potential gradient across the film is linear due to its relatively large area with a thin surface. Therefore, the magnitude of the electric potential can represent its gradient, which primarily acts as the driving force for electrically driven processes. By combining this relationship with the permittivity changes resulting from moisture loss, we can simulate the progression of residual potential throughout the drying process. The corresponding simulation results are shown in Fig. 5b.

Fig. 5 reveals that the residual potential at the start of drying is minimal, nearly zero, due to the high permittivity of the material. Following the FCC, the potential begins to increase gradually and accelerates significantly after the SCC. This simulation indicates that during the constant rate drying period, the residual potential is negligible, suggesting that the drying process is limited by external convection. In this scenario, the corona wind is responsible for the convective transport. These findings align with the current perspective that EHD drying operates primarily through a convective mechanism, as most current EHD drying studies focus on the constant rate drying period.

The residual potential becomes noticeable only upon entering the falling rate period, potentially triggering other electrically driven phenomena within the layer. This likelihood increases significantly after the SCC, as the potential rises more rapidly. This behaviour is particularly beneficial for the drying process, as it can enhance internal mechanisms during the falling rate period, a phase typically characterized by slow internal moisture migration.

### 3.4 Electric current

**Fig. 6:**
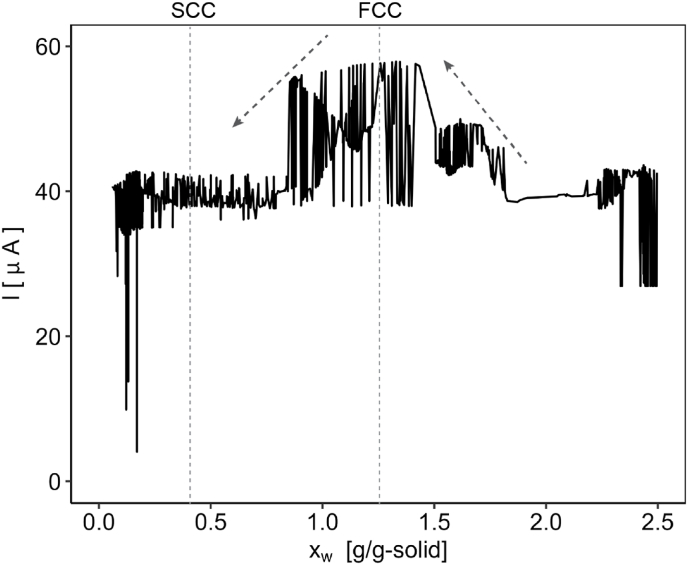
Electric current progression during EHD drying of starch thin layer using potential difference 17 kV at 20 °C.

We measured the current during EHD drying of starch and present the results in Fig. 6. On average, the current remained extremely low, giving values about 40 μA, which is typical for EHD processes. Throughout the measurement, we observed a consistent noise, resulting in a characteristic spiked pattern. Such patterns are common in corona discharge studies—known as corona pulses—and have been reported in previous research (Yin et al., 2014; Liu et al., 2014; Uckol and Ilhan, 2023).

Interestingly, we observed an unusual pattern. The current increased upon entering the falling rate period and then decreased again as drying progressed. This behaviour was consistently observed across multiple experiments in our lab under various drying conditions and with different materials (unreported experiments), regardless of the magnitude of the current. This suggests the occurrence of a noteworthy phenomenon during this period that merits further investigation.

Fig. 5 shows that the residual potential increases upon entering the falling rate period. This increase in potential may explain the rise in current observed in Fig. 6. However, instead of continuing to increase alongside the steep rise in potential after the SCC, the current decreased again.

**Fig. 7:**
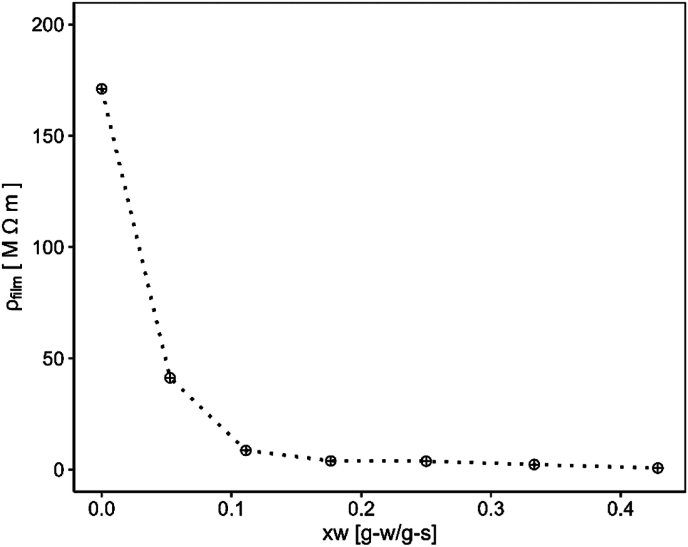
Resistivity of a starch layer measured as function of the moisture content.

In addition to the changes in permittivity in response to varying moisture content, the resistance of the material was also a moisture dependent. As shown in Fig. 7, the resistance of the starch layer increased rapidly as it dried. This rise in resistance likely contributed to the observed decrease in current as drying progressed beyond the falling rate period. Furthermore, the resistance may have become sufficiently high to prevent a further decrease in current upon entering the SCC phase.

This observation suggests that during the falling rate period both the increasing potential and increasing resistance occur simultaneously. Both may affect the driving force for electrically driven transport.

### 3.5 General discussion

In this study, we integrated multiple theoretical approaches with experimental characterizations and observations. This provided valuable insights into the property changes of thin layers during the EHD drying process. Furthermore, it enables us to mathematically model the progression of the electric potential throughout the drying process, offering a foundation for future research to couple this understanding with studies on electrically driven transport during EHD. Studying these phenomena *in situ* is challenging due to limitations of the use of instruments within the electric field. Conversely, ex situ investigations require boundary conditions related to the driving force, specifically the residual potential. This study thus bridges the gap by providing a framework that facilitates ex situ studies of internal transport during EHD drying, advancing the understanding of these complex processes.

The evolution of the volume fractions outlined in the previous chapter may serve as framework for relating electric potential during the drying process. This is highly influenced by the development of the layer itself. In this study, we applied a three-phase approach, which is generally applicable to most materials, particularly granular solids. However, certain mixtures may exhibit stronger interactions between phases, reducing the distinction between them. For instance, solutions like maltodextrin—commonly used in drying studies—demonstrate strong interactions between the solid and water and do not form distinct phases. Despite this, we believe the changes in properties during the falling rate period likely follow a similar trend to what is discussed in this study, although the specific mechanisms may vary.

Using the relationships presented in Fig. 5, Fig. 7, the energy dissipated through resistive (Ohmic) heating can be estimated based on Ohm's law. This energy can serve as an additional source for drying, complementing the evaporative cooling process. However, estimating the resulting temperature rise due to Ohmic heating is not straightforward, given the complexity of the underlying phenomena. More appropriate prediction requires coupling the Ohmic heating with mass and heat balance equations, which must be solved simultaneously.

## 4 Conclusions

In this study, we investigated the development of electric potential and current during EHD thin-layer drying, as dependent on the properties of the employed materials. Using starch granules as a model material, we examined the evolution of the layer's properties throughout the drying process and could predict the development of the electric potential over the product layer during EHD, by considering it to be a three-phase system comprising solid, water, and air.

Thin layer drying of starch by EHD follows a typical drying curve characterized by two critical points, marking the transitions from the constant rate period to the first and second falling rate periods. Once these critical points were identified, the evolution of the volume fractions for each component was modelled throughout the drying process. These volume fractions were subsequently used to estimate the layer's permittivity, which was then correlated with the evolution of the residual electric potential during drying. This provides a foundation for mathematically integrating electrically driven phenomena inside the drying product film during EHD.

Our analysis revealed a negligible electric potential during the constant rate period, indicating that drying is predominantly driven by convective transport from corona wind. As drying transitions into the falling rate period, this potential increases however, enabling electrically driven transport. This is counteracted by the rising resistance of the layer, creating a competitive effect.

Experimentally validating each step of the followed path remains a challenge. A key obstacle is the limited availability of instruments capable of functioning within an applied electric field. Achieving full validation would necessitate multidisciplinary research efforts and multiple ex situ investigations. Within the context of food process engineering, a practical form of validation could be derived from drying kinetics studies. This underscores the likelihood that a complete understanding and accurate characterization of the mechanisms governing EHD drying phenomena will require collaborative contributions from researchers across diverse disciplines.

## CRediT authorship contribution statement

Zulhaj Rizki: Conceptualization, methodology, investigation, formal analysis, visualization, writing – original draft.

Judith C. A. Ham: Conceptualization, methodology, investigation, writing – review and editing.

Remko Boom: Conceptualization, supervision, funding acquisition, writing – review and editing.

Maarten A.I. Schutyser: Conceptualization, supervision, funding acquisition, writing – review and editing.

## Declaration of competing interest

The authors declare the following financial interests/personal relationships which may be considered as potential competing interests:Maarten Schutyser and Remko Boom reports financial support was provided by 10.13039/501100003246Dutch Research Council. If there are other authors, they declare that they have no known competing financial interests or personal relationships that could have appeared to influence the work reported in this paper.

## Data Availability

The data is shared during the ‘Attach Files’ step. YODAModels and dataset underlying the publication - Material property changes during electrohydrodynamic (EHD) drying – a closer look into the falling rate period (Original data)

## Nomenclature

$\varepsilon_{r}$: relative permittivity [dimensionless]
*m*: mass of each component in the layer [g]
ν: volume fraction of each component in the layer [dimensionless]
ρ: density of component [$g/cm^{3}$]
V: partial volume of each component [$cm^{3}$]
$x_{w}$: moisture content dry basis [$g_{w}/g_{s}$]

**Subscripts**
a: air
f: film layer
s: solid
w: water
